# Tomatidine inhibits porcine epidemic diarrhea virus replication by targeting 3CL protease

**DOI:** 10.1186/s13567-020-00865-y

**Published:** 2020-11-11

**Authors:** Pengcheng Wang, Juan Bai, Xuewei Liu, Mi Wang, Xianwei Wang, Ping Jiang

**Affiliations:** 1 Key Laboratory of Animal Disease Diagnostics and Immunology, Ministry of Agriculture, MOE International Joint Collaborative Research Laboratory for Animal Health & Food Safety, College of Veterinary Medicine, Nanjing Agricultural University, Nanjing, 210095 China; 2 Jiangsu Co-Innovation Center for Prevention and Control of Important Animal Infectious Diseases and Zoonoses, Yangzhou, China

**Keywords:** tomatidine, PEDV replication, 3CL protease

## Abstract

Porcine epidemic diarrhea virus (PEDV) causes lethal diarrhea in suckling piglets, leading to severe economic losses worldwide. There is an urgent need to find new therapeutic methods to prevent and control PEDV. Not only is there a shortage of commercial anti-PEDV drugs, but available commercial vaccines fail to protect against highly virulent PEDV variants. We screened an FDA-approved library of 911 natural products and found that tomatidine, a steroidal alkaloid extracted from the skin and leaves of tomatoes, demonstrates significant inhibition of PEDV replication in Vero and IPEC-J2 cells in vitro. Molecular docking and molecular dynamics analysis predicted interactions between tomatidine and the active pocket of PEDV 3CL protease, which were confirmed by fluorescence spectroscopy and isothermal titration calorimetry (ITC). The inhibiting effect of tomatidine on 3CL protease was determined using cleavage visualization and FRET assay. Tomatidine-mediated blocking of 3CL protease activity in PEDV-infected cells was examined by western blot detection of the viral polyprotein in PEDV-infected cells. It indicates that tomatidine inhibits PEDV replication mainly by targeting 3CL protease. In addition, tomatidine also has antiviral activity against transmissible gastroenteritis virus (TGEV), porcine reproductive and respiratory syndrome virus (PRRSV), encephalo myocarditis virus (EMCV) and seneca *virus A* (SVA) in vitro. These results may be helpful in developing a new prophylactic and therapeutic strategy against PEDV and other swine disease infections.

## Introduction

PEDV, an enveloped, positive-sense, single-stranded RNA virus, is a member of the Coronavirinae subfamily [1, 2], which comprises viruses that cause a variety of diseases in mammals and birds, ranging from enteritis in cows and pigs, to upper respiratory disease in chickens, and potentially lethal human respiratory infections, such as severe acute respiratory syndrome (SARS) [3], Middle East respiratory syndrome (MERS) [4], and the novel Coronavirus Disease 2019 (COVID-19) [5]. Fecal–oral transmission is believed to be the main mode of PEDV transmission [6]. The latest research indicates that airborne transmission may also contribute to a PEDV outbreak [7], similar to SARS-CoV-2 and MERS-CoV. Owing to antigenic, genetic (> 10% amino acid variation between respective S-proteins) and phylogenetic (G1 vs G2) differences between vaccine and field epidemic strains, current PEDV vaccines appear to have low to moderate efficacy [8]. There is a need for alternative approaches to control this disease, such as effective antiviral drugs for PEDV treatment.

The interaction between PEDV and pigs has been well studied. Several host antiviral factors, including the bone marrow stromal cell antigen 2 (BST2) [9], interleukin-11 (IL-11) [10], interferon-lambda (IFN-lambda) [11], GTPase-activating protein-binding protein 1 (G3BP1) [12], and cholesterol 25-hydroxylase (CH25H) [13], have been reported to show antiviral activity against PEDV infection. Some natural compounds and compositions have also been reported to show anti-PEDV activity in vitro, such as Griffithsin [14], Coumarin [15], and prenylated phenolic compounds [16]. However, the mechanism of antiviral activity is not well understood, and there are no commercial anti-PEDV drugs available to the pig breeding industry. In this study, we screened a library of 911 natural products and found that tomatidine, a steroidal alkaloid that can be extracted from the skin and leaves of tomatoes [17], significantly inhibited the replication of PEDV by directly inhibiting 3CLpro activity, and showed antiviral activity against other swine disease viruses as well, demonstrating excellent potential as a natural broad-spectrum antiviral product.

## Materials and methods

### Cells, viruses and reagents

Vero cells, ST cells, Marc-145 cells, BHK-21 cells, and IPEC-J2 cells were maintained in Dulbecco’s modified Eagle’s medium (DMEM) (Gibco, USA) with 10% fetal bovine serum (Lonsera, Uruguay), penicillin (250 U/mL), and streptomycin (250 ug/mL). The cells were incubated at 37 °C in a humidified incubator with 5% CO_2_. PEDV MS (GenBank accession no. MT683617), YZ (GenBank accession no. MK841495.1), SH (GenBank accession no. MK841494.1) [18], and CV777 (GenBank accession no. AF353511.1) strains were maintained in our laboratory and passaged in Vero cells with 2.5% trypsin. The MS, YZ, and SH strains belong to variant strains; CV777 belongs to a classical strain. The MS strain was used for all experiments and is represented by “PEDV” in this article. TGEV JS2012 (GenBank accession no. KT696544.1) passaged in ST cells, the highly pathogenic PRRSV strain BB0907 (GenBank accession no. HQ315835.1) passaged in Marc-145 cells, EMCV NJ08 (GenBank accession no. HM641897), and SVA CH-SD (GenBank accession no. MH779611.1) passaged in BHK-21 cells, were maintained in our laboratory. Tomatidine with purity > 99% was used for in vitro experiments (Selleck Chemicals, USA).

### Screening of natural product library

**Figure 1:**
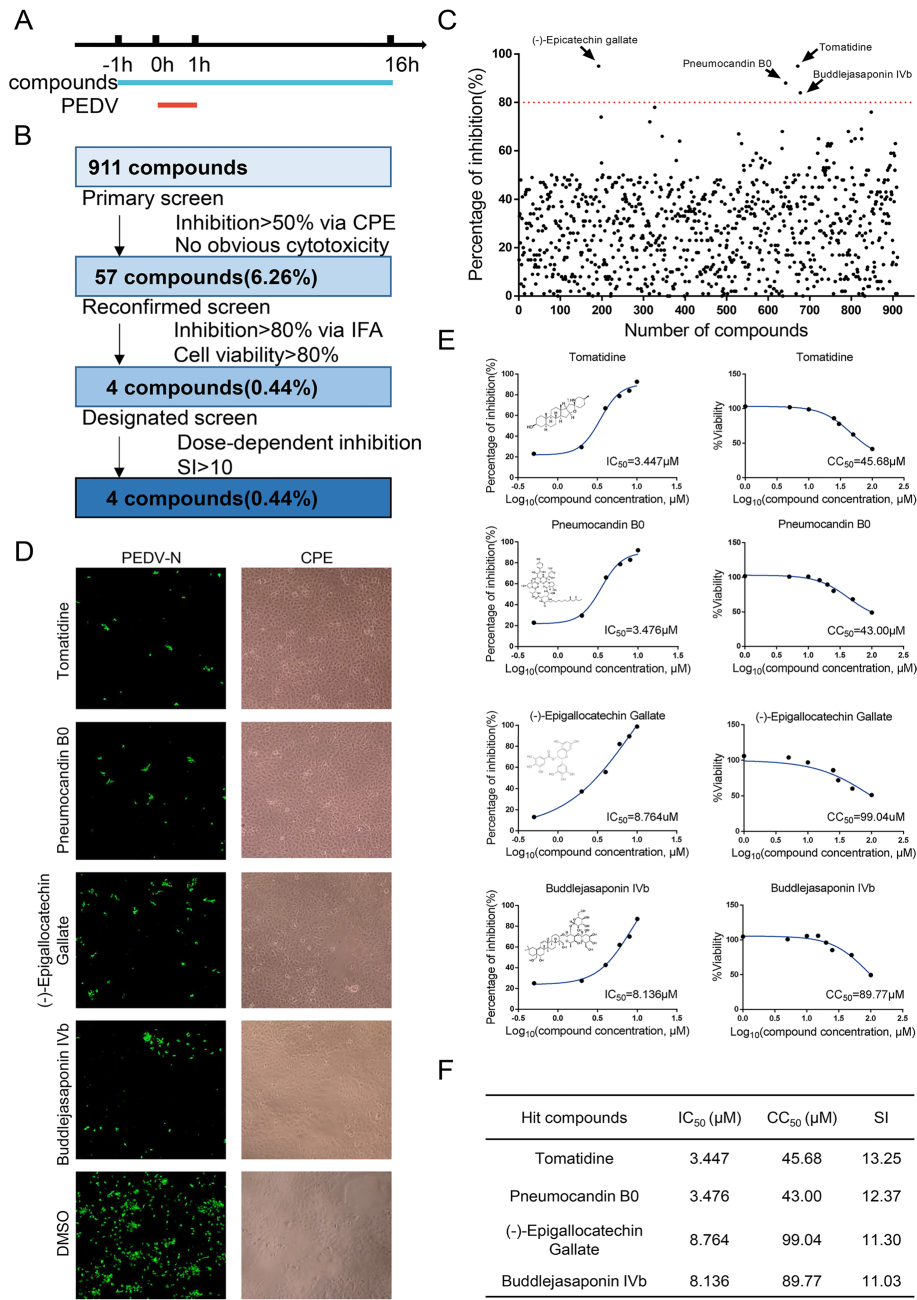
**Screening protocol for PEDV inhibitors. A** Screening procedure. Vero cells were treated with 10 μM compound for 1 h, then infected with PEDV (0.01 MOI) for 1 h. The cells were washed with PBS, then incubated in medium containing 10 μM compound for another 15 h. **B** Screening process flowchart. The criteria for passing the primary screening were that the compound must have no apparent cytotoxicity and must reduce CPE by at least 50% compared with the DMSO treatment. The criteria for passing the secondary screening were that the compound must leave cells at least 80% viable and inhibit PEDV by more than 80%. Compounds that passed the third screen inhibited PEDV in a dose-dependent manner and had a selective index (SI) higher than 10. **C** Each dot represents the percent inhibition of each compound. The dots located above the dotted line indicate 80% or greater inhibition. **D** IFA of infected cells treated with one of the four designated compounds. PEDV N-protein is colored green; a bright field shows CPE. **E** IC_50_ and CC_50_ curves of the four designated compounds. Cell viability is calculated as a percentage of the viability in the cells treated with the compounds divided by that in the DMSO-treated cells. The structure of each compound is inset. **F** SIs of the four designated compounds.

A library (FDA approved) of 911 natural products was purchased from Selleck Chemicals. These compounds were stored as 10 mM stock solutions in DMSO at − 80 °C until use. The workflow for high-throughput screening (HTS) of the library was carried out as shown in Figure 1A and B. Vero cells were seeded in 96-well plates at 5 × 10^4^ cells per well. When approximately 90% confluent, the cells were treated with 10 μM compound or DMSO (1μL, 0.2% v/v) for 1 h and then infected with PEDV (0.01 MOI) or mock infected for 1 h. The cells were then washed with PBS, then culture medium containing 10 μM compound was added back to each well. At 16 hpi, CPE and IFA were observed under a microscope. The fluorescence intensity of IFA was measured by ImageJ software. The percentage of inhibition of fluorescence intensity is calculated by drug treatment relative to DMSO treatment.

During primary screening, compounds were ruled out if they resulted in any visible cytotoxicity or demonstrated less than 50% reduction of CPE compared with the DMSO control group. For the second round of screening, cell viability had to be 80% or greater, and the inhibition of PEDV had to be 80% or greater as determined by IFA. The IC_50_ (concentration of compounds was 0.5, 2.0, 4.0, 6.0, 8.0, and 10.0 μM) and CC_50_ (concentration of compounds was 1.0, 5.0, 10.0, 25.0, 30.0, 50.0, and 100.0 μM) of each remaining candidate compound were calculated using log (inhibitor) vs. response—Variable slope (four parameters) method by Graphpad Prism 6.0 software (GraphPad Software, CA, USA), and those that displayed dose-dependent inhibition of PEDV and selectivity index (SI, SI = CC_50_/IC_50_) over 10 were considered for further study. In addition, cell viability was tested using an enhanced Cell Counting Kit-8 (CCK-8) (Beyotime, China) following the manufacturer’s instructions. The CC_50_ was calculated using GraphPad Prism 6.0 software. DMSO was used as the negative control.

### PEDV infectivity inhibition assay

An IFA was used to examine the effect of tomatidine on PEDV infection in Vero cells. Serially diluted tomatidine was added to the culture medium of the cells (final concentrations were 0.5, 2, 4, 6, 8, and 10 μM). DMSO was used as the negative control. PEDV (0.01 MOI) was then added to the cells, and cultures were incubated for 16 h at 37 °C. The cells were then fixed with 4% paraformaldehyde for 20 min, washed with PBS, and then permeabilized with 0.1% Trition X-100 for 20 min at 37 °C. The treated cells were incubated with a mouse anti-PEDV N-protein mAb prepared in our laboratory (1:200 dilution) for 2 h at 37 °C. The cells were then washed three times with PBS and incubated with FITC-labeled goat anti-mouse IgG (H + L) antibody (1:200 dilution) (A0568, Beyotime, China) at 37 °C for 1 h. After washing with PBS, the cells were visualized using a Zeiss inverted fluorescence microscope. The integrated optical density of fluorescence was determined using ImageJ software.

### Western blot assay

The cells were lysed with 100 μL of RIPA lysis buffer (Beyotime, China) on ice for 15 min, then resolved by SDS-PAGE and transferred to a nitrocellulose membrane. After transfer, the membrane was incubated in blocking buffer (5% non-fat milk in PBST w/v) for 2 h at room temperature, washed three times with PBST, then probed with the following antibodies: anti-PEDV N-protein (1:1000), anti-PRRSV N-protein (1:1000), anti-TGEV N-protein (1:1000), anti-EMCV VP1-protein (1:1000), and anti-SVA VP1-protein (1:1000), all prepared in our laboratory; anti-GAPDH (60004-1-Ig, 1:5000; Proteintech Group, USA), anti-β-actin (1:1000; sc-47778, Santa Cruz, USA), anti-GFP (1:2000; 66002-1-Ig, Proteintech Group, USA), anti-Flag (1:5000; 20543-1-AP, Proteintech Group, USA), and anti-His (1:2000; AF5060, Beyotime, China) for 2 h at room temperature, and then washed three times with PBST. The membranes were incubated with HRP-conjugated goat anti-mouse and anti-rabbit IgG (H–L) secondary antibodies (1:1000; A0216, A0208, Beyotime, China). Bound proteins were exposed with an ECL Kit (Tanon, China).

### RNA extraction and quantitative real-time PCR

Total RNA was extracted from the Vero, Marc-145, ST, BHK-21, and IPEC-J2 cells using a Total RNA Kit (Omega Bio-tek, USA) and reverse transcribed into cDNA using HiScript qRT SuperMix (Vazyme, China), following the manufacturer’s instructions. The following primers were used: PEDV N (Forward-5′-TTCTTGTTTCACAGGTGGATG-3′, Reverse-5′-GCTGCTGCGTGGTTTCA-3′); EMCV VP1 (Forward-5′-CCCCACCTCTGCTAAGATACTAAC-3′, Reverse-5′-TGGGACTGGACCTATCATAGAAG); monkey GAPDH (Forward-5′-CCTTCCGTGTCCCTACTGCCAAC-3′, Reverse-5′-GACGCCTGCTTCACCACCTTCT-3′); and porcine GAPDH (Forward-5′-TGGTGAAGGTCGGAGTGAAC-3′, Reverse-5′-AGTGGAGGTCAATGAAGGGG-3′). The detection of mRNA levels of TGEV, PRRSV, and SVA was performed as described previously [13, 19, 20]. Quantitative RT-PCR was performed using AceQ^®^ qPCR SYBR^®^ Green Master Mix (Vazyme, China). Each reaction was performed in triplicate and results are expressed as mean ± standard deviation (SD).

### Virus titration

Vero cells grown in 96-well plates were infected with tenfold serial dilutions of PEDV samples in four replicates. After 1 h at 37 °C, the culture medium was replaced with fresh DMEM. The plates were incubated for 48–72 h at 37 °C. ST cells grown in 96-well plates were infected with tenfold serial dilutions of TGEV samples in four replicates. After 1 h at 37 °C, the culture medium was replaced with fresh DMEM. The plates were incubated for 48–72 h at 37 °C. Marc-145 cells grown in 96-well plates were infected with tenfold serial dilutions of PRRSV samples in four replicates. After 1 h at 37 °C, the culture medium was replaced with fresh DMEM. The plates were incubated for 72-96 h at 37 °C. BHK cells grown in 96-well plates were infected with tenfold serial dilutions of EMCV/SVA samples in four replicates. After 1 h at 37 °C, the culture medium was replaced with fresh DMEM. The plates were incubated for 48–72 h at 37 °C. Virus titers are expressed as TCID_50_, calculated using the Reed-Muench method.

### Inactivation assay

Tomatidine (10 μM) or DMSO was incubated with PEDV (0.01 MOI) at 37 °C for 3 h and 5 h. A mixture of tomatidine (10 μM) or DMSO and PEDV was placed into Vero cells seeded in 24-well plates. After incubation at 37 °C for an additional 1 h, the culture supernatants were replaced with fresh DMEM and incubated for an additional 12 h (Figure 3A). The cells were then washed with PBS, and the mRNA levels of PEDV N and GAPDH in the cells were measured using qRT-PCR.

### Virus attachment assay

Vero cells were pretreated with tomatidine (10 μM) or DMSO for 1 h at 37 °C, and then infected with PEDV (0.01 MOI) for the time indicated (15 min, 30 min, 1 h) at 4 °C (Figure 3B). The cells were then washed with ice-cold PBS, and the mRNA levels of PEDV N and GAPDH in the cells were measured using qRT-PCR.

### Virus internalization assay

Vero cells were infected with PEDV (0.01 MOI) at 4 °C for 1 h. The supernatant was replaced with DMEM containing tomatidine (10 μM) or DMSO, and then incubated at 37 °C for the time indicated (30 min, 1 h, 2 h) (Figure 3C). The cells were washed with citrate buffer (pH 3) to remove non-internalized virus. The mRNA levels of PEDV N and GAPDH in the cells were then measured using qRT-PCR.

### Virus replication assay

Vero cells were incubated with PEDV (0.01 MOI) at 37 °C for 1 h and washed three times with PBS to remove free virus. At 4 hpi, the culture medium was replaced with fresh DMEM containing tomatidine (10 μM) or DMSO, and the cultures were incubated at 37 °C (Figure 3D). The mRNA levels of PEDV N and GAPDH in the samples collected at 6, 8, 10 hpi were measured using qRT-PCR.

### Virus release assay

Vero cells were infected with PEDV (0.01 MOI) for 1 h at 37 °C. The culture medium was then replaced with fresh DMEM. At 10 hpi, the cells were washed three times with PBS and the culture medium was replaced with fresh DMEM containing tomatidine (10 μM) or DMSO. The cultures were incubated at 37 °C for 0.5, 1, and 2 h, at which time the supernatants were harvested (Figure 3E). The mRNA levels of PEDV N in the released virus was quantified by absolute fluorescence quantification.

### In silico docking

The crystal structure of PEDV nsp5 was obtained from the Protein Data Bank (3CLpro, PDB:4XFQ). Due to lack of crystal structure, the protein sequences of nsp3 (PLP2), nsp12 (RdRp), nsp13 (NTP), nsp14 (ExoN), nsp15 (NendoU), and nsp16 (2′-o-methyltransferase) were subjected to comparative homology modeling using SWISS MODEL, which has been widely used and cited by more than 470 articles, to generate a putative 3D model. SWISS MODEL performs the sequence alignments and searches the putative template protein to generate a 3D model for a query sequence. All the modeling parameters were set to default. The three-dimensional structure of tomatidine was obtained from PubChem (Compound CID: 65576).

The Autodock 4.2 program was used for the docking of tomatidine to the active pocket of potential replication-relevant proteins. We generated a grid map around selected active site residues with grid point spacing of 0.375 Å. The potential tomatidine-protein complex was generated by genetic algorithm using the default parameters. The Estimated Free Energy of Binding was ranked, and the top two complexes were employed for further MD analysis. The docking results were visualized using PyMOL 2.3.2.

### Molecular dynamics simulations

The ligand bound to the GROMOS87/GROMOS96 force field generated by PRODRG 2.5. Simulations were conducted with the Gromacs package using the GROMOS96 43A1 force field. The simple point charge 216 (SPC 216) model of water was used to solvate the protein in a periodic dodecahedron box extending 10 Å from the nearest protein atom. The solvated system was then neutralized with Na+ and Cl− ions, minimized by the steepest descent method (50 000 steps), and equilibrated with a 100-ps constant volume (NVT) simulation. The production runs were conducted in a constant pressure ensemble (NPT). The temperature was set to 300 K and controlled with V-rescale. Long-range electrostatic interactions were treated with the particle-mesh Ewald (PME) method. The pressure was coupled to 1 bar using the Parrinello-Rahman method. All bond lengths were constrained with the LINear Constraint Solver (LINCS) algorithm. A cut-off of 14 Å was used to calculate short-range van der Waals and electrostatic interactions. The time step was 2 fs and the total simulation time was 10 ns. RMSD, distance, and the number of hydrogen bonds between tomatidine and active pockets of the proteins were analyzed to judge binding stability and convergence.

### Prokaryotic expression and purification of PEDV 3CL protease

The pET-32a-3CLpro was transformed into *E. coli* strain BL21, and the cells were cultured at 37 °C in LB medium. When optical density at 600 nm (OD600) reached 0.8, the culture was cooled to 27 °C and supplemented with 1 mM IPTG. The cells were harvested after incubation at 27 °C for 7 h, resuspended in PBS and disrupted by ultrasonication. The supernatant was filtered and loaded onto Ni-Sepharose (GE Healthcare, USA). Finally, the His-tagged protein was eluted using a linear gradient between the binding buffer and elution buffer A (20 mM Tris, pH 7.4, 500 mM NaCl, and 250 mM imidazole). Low concentration imidazole (50 mM) was used to wash impurities, and high concentration imidazole (250 mM) was used to elute targeted protein. The target protein was condensed and desalinated using Amicon Ultra-4 (30 kDa, GE Healthcare, USA). The proteins were analyzed by SDS-PAGE. All of the purification procedures were performed at 4 °C to avoid unexpected degradation.

### Fluorescence quenching analysis

The fluorescence quenching assay was measured by a PerkinElmer EnSpire Multimode Plate Reader. The reaction medium (200 μL) contained 190 μL of 3CLpro solution at the concentration of 1 μM and 10 μL of tomatidine with different final concentrations (0, 30, 60, 90, 120, and 150 μM). Following incubation at room temperature for 15 min, the fluorescence spectra of 3CLpro with the different concentrations of tomatidine were recorded in the wavelength range of 300–500 nm upon excitation at 280 nm.

The Stern–Volmer equation was used to describe fluorescence quenching as follows: F_0_/F = 1 + K_q_τ_0_[Q] = 1 + K_sv_[Q]. In this equation, F_0_ and F represent the fluorescence intensities in the absence and presence of tomatidine. τ_0_ (10^−8^s) indicates the lifetime of the fluorophore without quencher. K_q_ is the bimolecular quenching constant. [Q] refers to the concentration of the quencher, and K_sv_ is the Stern–Volmer quenching constant. Hence, the above equation may be applied to determine K_sv_ by linear regression of a plot of F_0_/F versus [Q]. Each measurement was repeated three times.

### ITC

All measurements were performed using the MicroCal iTC200 calorimeter in an ITC buffer (20 mM Tris, pH 7.4, 500 mM NaCl, pH 8.2) while stirring at 700 rpm. The stock solution of compound and 3CLpro protein were diluted with the ITC buffer to a compound concentration of 300 μM and a protein concentration of 10 μM before titrations. The final concentration of DMSO in the reaction buffer was less than 2% of the total volume. Protein solution was titrated using the compound. All titrations were performed using an initial injection of 0.4 μL followed by 19 identical injections of 2 μL with a duration of 4 s and an interval of 120 s between injections. The data were analyzed by MicroCal iTC200 software. The last three data points were averaged and subtracted from each titration to account for the heat of dilution. Each measurement was repeated three times.

### Plasmid construction

For PEDV 3CLpro eukaryotic and prokaryotic expression plasmids, the coding sequence for 3CLpro was reverse transcribed and amplified from PEDV MS strains. The resulting PCR products were assembled into pCAGGS-Flag and pET-32a. Mutagenesis of PEDV 3CLpro constructs were carried out by overlap extension PCR using specific mutagenic primers. The plasmids were verified by sequencing and double enzyme digestion.

For functional substrates of PEDV 3CLpro, a nucleotide sequence encoding for an 8-amino-acid stretch corresponding to the cleavage site of PEDV 3CLpro derived from the junction of the nsp5 and nsp6 genes (YGVNLQ^SG) in the PEDV genome was inserted into the coding sequence of GFP between amino acids G190 and D191. The first PCR product (N-GFP) was amplified with a reverse primer harboring the N-terminal of the cleavage site coding sequence (YGVNLQ). The second PCR product (C-GFP) was amplified with a forward primer harboring the C-terminal of the cleavage site coding sequence (VNLQSG). The first and second PCR products were assembled using overlap PCR and cloned into pCAGGS.

### Cleavage visualization

Vero cells cultured in 24-well plates were co-transfected with 250 ng/well of GFP_nsp5/6_ encoding plasmid, 250 ng of 3CLpro expressing plasmid, or an empty vector. At 12 hpi, the culture supernatants were replaced with fresh DMEM containing the indicated concentrations (10, 20, 30 μM) of tomatidine or DMSO. At 24 hpi, the cells were harvested and the cleaved fragment of GFP_nsp5/6_ was visualized using western blot.

### FRET-based assays for enzymatic characteristics

The polypeptide substrate Dabsyl-YNSTLQ↓AGLRKM-E-Edans was chemically synthesized by the GenScript Corporation. PEDV 3CL protease was used at final concentrations of 0.5, 1, and 1.5 μM; and FRET peptide was added to the protein in a black 96-well plate at final concentrations of 10 μM. PRRSV GP5 protein expressed and purified at the same time with 3CL protease was used as a negative control at final concentrations of 10 μM. The mixtures were then further incubated at 37 °C for 20 min, and fluorescence was monitored at 340 nm excitation and 485 nm emissions every minute.

Obacunone was used as a control drug. It is an FDA-approved compound with molecular weight similar to tomatidine, but it has no antiviral activity on PEDV (according the data from HTS). Tomatidine (25, 50 μM), obacunone (50 μM, negative control) or DMSO were pre-incubated with 1 μM 3CLpro for 20 min at 37 °C, and 10 μM FRET peptide was added to the mixture in a black 96-well plate [21]. The mixtures were then further incubated at 37 °C for 20 min, and fluorescence was monitored at 340 nm excitation and 485 nm emissions every minute. The mock represents FRET peptides with no protease. The relative fluorescence units (RFU) of the experimental group were obtained after subtracting fluorescence readings of the mock. The percentage of inhibition was calculated as follows: percentage of inhibition (%) = 100 × [1 − RLU of the tomatidine group (20–0 min)/RLU of the negative control group (20–0 min)] (20).

### Preparation of anti-3CLpro serum

All procedures involving animals for this study were approved by and performed in accordance with the Animal Ethics Committee and Nanjing Agricultural University animal experiment central guidelines, respectively. Female 6–8 week-old BALB/c mice were immunized with 50 μg of purified 3CLpro protein in 0.2 mL, emulsified in the same amount of Freund’s complete adjuvant. Two booster injections were administered with equal amounts of immunogen and Freund’s incomplete adjuvant at 3-week intervals. Ten days after the last injection, antiserum was collected.

### Passage of PEDV under tomatidine pressure in vitro

PEDV was cultured and passaged by 10 passages in IPEC-J2 cells in the presence of tomatidine at an increasing concentration of 2–4–6 μM or in DMSO. The total RNA was extracted from the virus cultures, tomatidine-F10, DMSO-F10, and F0. The 3CLpro gene was amplified and sequenced by using the primers located at ~ 150 bp upstream and downstream of 3CLpro. To assess the sensitivity of F10 viruses to tomatidine, IPEC-J2 cells were inoculated with the virus (tomatidine-F10, DMSO-F10, and F0) at 0.1 MOI, and the culture medium was replaced with fresh DMEM containing tomatidine (10 μM) or DMSO at 4 hpi. The mRNA levels of PEDV N and GAPDH in the samples collected at 16 hpi were measured using qRT-PCR.

### Broad-spectrum antiviral assessment

Western blot, qPCR, and TCID_50_ were used to examine tomatidine’s inhibition of other swine disease viruses. Three designated compound concentrations were added to the culture medium (final concentrations were 2.5, 5, and 10 μM). DMSO was used as the negative control. PRRSV (0.01 MOI), TGEV (0.01 MOI), and SVA/EMCV (0.01 MOI) were then inoculated in Marc-145, ST, and BHK-21 cells, respectively. The proteins were harvested at 48 h, 24 h, and 18 h post-inoculation.

### Statistical analysis

Statistical analysis was performed using GraphPad Prism6 software. Results are expressed as mean ± standard deviation (SD). Differences between groups were examined for statistical significance using one-way or two-way analysis of variance (ANOVA). The asterisks in the figures indicate significant differences (*P < 0.05; **P < 0.01; ***P < 0.001; ns = not significant).

## Results

### Library screening

Vero cells were treated with 10 µM natural product compounds, and infected with PEDV as detailed in the timeline (Figure 1A). The results indicated that 57 (6.26%) compounds showed no apparent cytotoxicity, and reduced cytopathic effect (CPE) by 50% compared with DMSO alone. These compounds were then subjected to a second round of screening (Figure 1B). Four of the compounds produced negligible cytotoxicity and inhibited PEDV infection by over 80% as determined by indirect immunofluorescence assay (IFA) (Figure 1C and D). In addition, they inhibited PEDV in a dose-dependent manner and had a selectivity index (SI) greater than 10 (Figure 1E and F). Tomatidine was chosen for further study as it had the highest SI and the lowest price.

**Figure 2:**
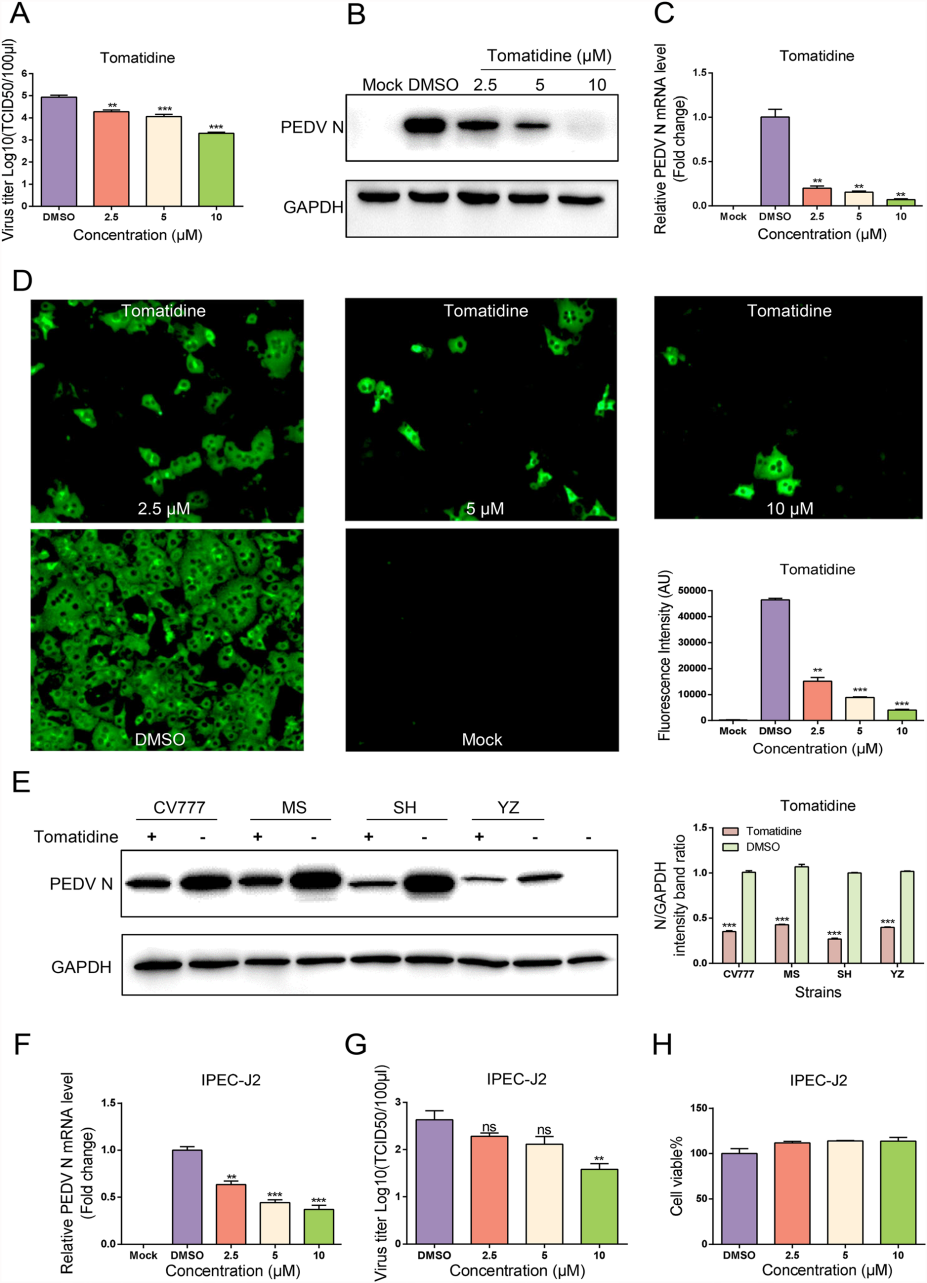
**Identification of anti-PEDV activity of tomatidine in Vero and IPEC-J2 cells.** Vero cells were pretreated with the indicated concentrations of tomatidine (**A**–**D**) for 1 h, and then infected with PEDV for 1 h at 37 °C. The cells were washed with PBS, then incubated in fresh medium containing tomatidine for 16 h. DMSO served as the treatment control. **A** Culture supernatants were collected at the indicated time points for viral titration. Results are expressed as TCID_50_. Titers from three independent experiments are shown as mean ± SD (error bars). **B** Western blot of N-protein in cells infected with PEDV and treated with the indicated concentrations of tomatidine or DMSO, at 16 hpi. **C** Relative PEDV N mRNA levels, determined by qRT-PCR, and expressed relative to that in DMSO-treated cells. The internal loading control was GAPDH. **D** Light microscope and IFA images of Vero cells infected with PEDV and treated with tomatidine, at 16 hpi. Viral N-protein is green. **E** Western blot of N-protein in cells infected with different PEDV genotypes and treated with indicated concentrations of tomatidine or DMSO, at 16 hpi. **F**, **G** IPEC-J2 cells were pretreated with tomatidine for 1 h, and infected with PEDV for 1 h at 37 °C. After incubation for a total of 24 h in fresh medium containing tomatidine, the culture supernatants were collected at the indicated time points for viral TCID_50_ titration, and the relative PEDV N mRNA levels in the cells were determined by qRT-PCR as above. **H** Viability of cells pretreated with the indicated concentrations of tomatidine and incubated for 24 h in medium containing tomatidine. The results are from one of three independent experiments. Error bars represent the SD. The asterisks in the figures indicate significant differences (*P < 0.05; **P < 0.01; ***P < 0.001; ns: not significant).

To determine the dose range of tomatidine having anti-PEDV activity, Vero cells were treated with 2.5, 5, and 10 μM tomatidine for 1 h and then infected with PEDV. Median Tissue Culture Infectious Dose (TCID_50_), Western blot and qRT-PCR analysis showed that the virus titers, N-protein, and mRNA levels decreased in a dose-dependent manner (Figure 2A–C). At 16 h post-infection (hpi), IFA showed that the number of infected cells in the tomatidine-treated groups were obviously lower than those in the negative control (Figure 2D). In addition, tomatidine had similar inhibitory effects on the different PEDV variant strains tested (YZ, MS, and SH), as well as on the classic CV777 strain (Figure 2E).

To understand the anti-viral activity of tomatidine in porcine cells, IPEC-J2 cells were treated with 2.5, 5, and 10 μM tomatidine for 1 h and then infected with PEDV (0.1 MOI). TCID_50_ and qRT-PCR analysis showed that the virus titers and ORF7 mRNA levels significantly decreased in a dose-dependent manner (Figures 2F, G). Meanwhile, the IPEC-J2 cells indicated that the tomatidine displayed no cytotoxicity (Figure 2H).

### Effect of tomatidine on viral inactivation, attachment, entry, replication, and release

**Figure 3:**
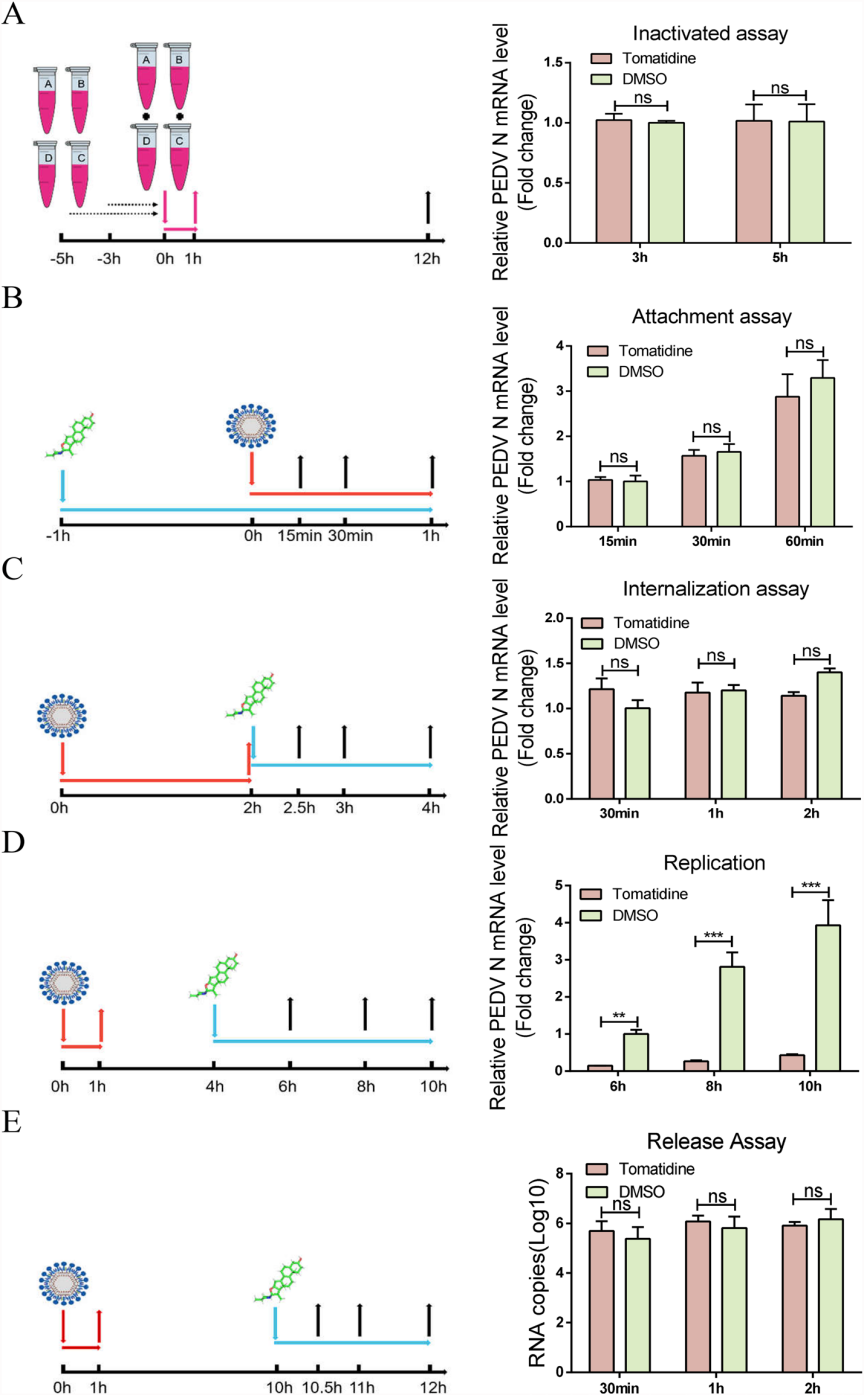
**Effect of tomatidine on the inactivation, attachment, entry, replication, and release of PEDV. A** Inactivated assay. Four groups: A. PEDV (10 μM) + tomatidine, B. PEDV (0.01 MOI) + DMSO, C. tomatidine (10 μM), and D. DMSO, were prepared and incubated at 37 °C for 3 h. Group A was then mixed with group D, and group B was mixed with group C, and the mixtures were added into Vero cells seeded in 24-well plates. After incubation at 37 °C for another 1 h, culture supernatants were replaced with fresh DMEM and incubated for an additional 12 h. The cells were washed with PBS, and the mRNA levels of PEDV N and GAPDH in the cells were measured using qRT-PCR. **B** Virus attachment assay. **C** Virus internalization assay. **D** Virus replication assay. **E** Virus release assay. The results are from one of three independent experiments. Error bars represent the SD. The asterisks in the figures indicate significant differences (*P < 0.05; **P < 0.01; ***P < 0.001; ns: not significant).

To further explore the mechanism by which tomatidine inhibits PEDV infection, we first tested whether tomatidine is directly virucidal and kills PEDV particles. As shown in Figure 3A, tomatidine treatment failed to directly inactivate PEDV. Next, the relative amount of PEDV N mRNA to GAPDH was detected by qRT-PCR to determine the effect of tomatidine on PEDV attachment, internalization, and replication. The results showed that tomatidine treatment prior to PEDV infection did not significantly block virus attachment to Vero cells, indicating that tomatidine does not inhibit PEDV attachment to cells (Figure 3B). In evaluating the effect of tomatidine on PEDV internalization, as shown in Figure 3C, we determined that tomatidine treatment accompanied by virus internalization did not significantly impede virus replication compared to treatment with DMSO. We then examined the effect of tomatidine on PEDV replication by adding tomatidine during the replication stage. As shown in Figure 3D, tomatidine treatment decreased PEDV N mRNA levels by approximately tenfold compared to treatment with DMSO, suggesting that tomatidine inhibits PEDV replication. In addition, the virus release assay showed that there was no significant difference in PEDV RNA levels in the supernatants between the treatment with tomatidine and DMSO (Figure 3E). Taken together, these results indicate that tomatidine inhibits PEDV infection primarily by affecting viral replication.

### In silico tomatidine is predicted to target the active pocket of PEDV 3CL protease

**Figure 4:**
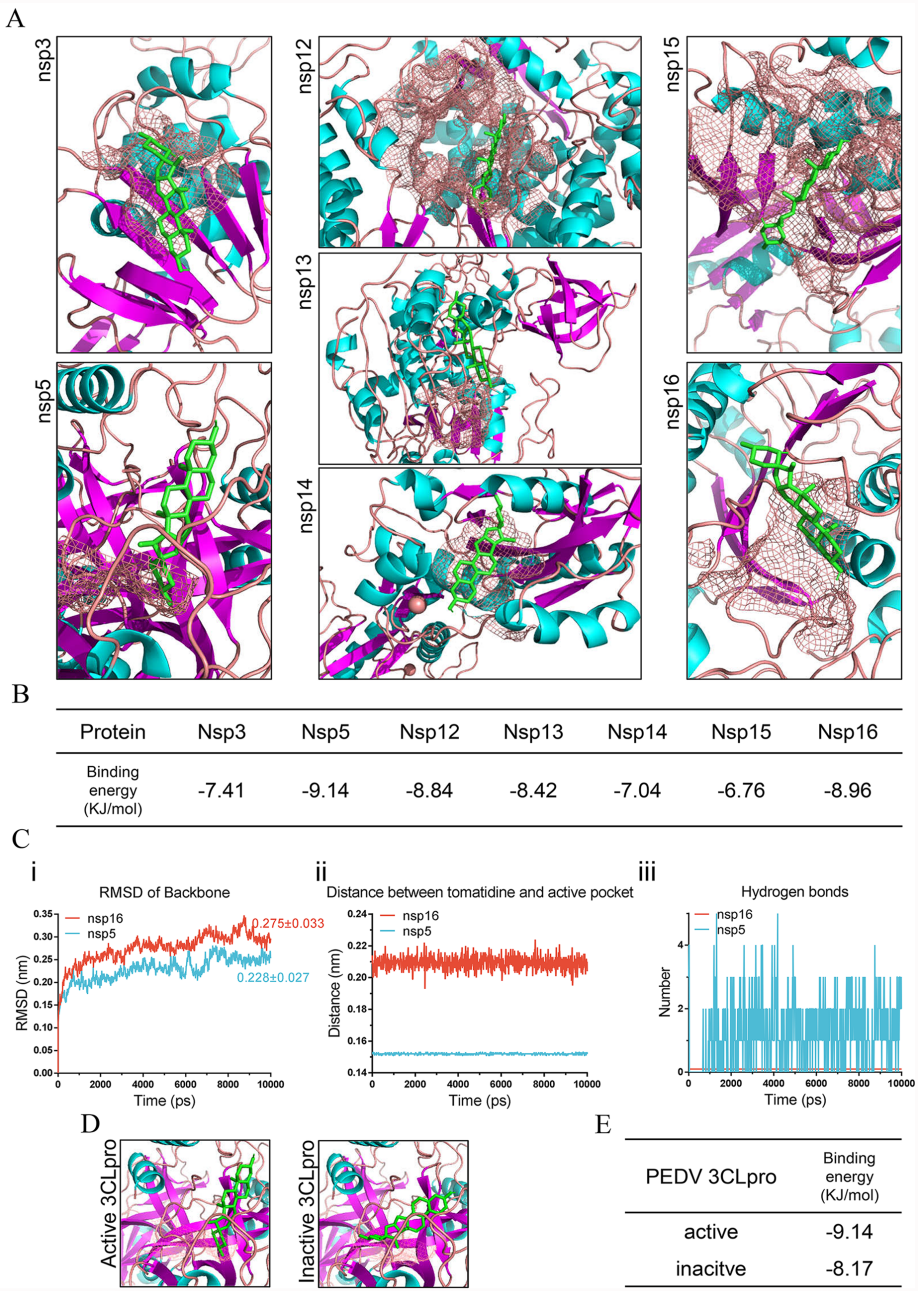
**In silico tomatidine targeted the active pocket of PEDV 3CL protease. A** Docked conformations of tomatidine with PEDV nsp3 PLP2, nsp5 3CLpro, nsp12 RdRp, nsp13 NTP, nsp14 ExoN, nsp15 Nendo U, and nsp16 2′-o-methyltransferase. The compounds and proteins are represented as sticks and cartoons, respectively. The compounds are colored green. The proteins are colored according to their secondary structures (helix = blue, sheet = purple, loop = pink). The active sites of enzyme pockets are shown as a mesh. **B** The binding energy of the tomatidine–protein complex, calculated using Autodock, is listed. **C** Overall dynamic behaviors in the MD simulations. (i) RMSD of backbones of nsp5 (red) and nsp16 (blue); (ii) Distance between tomatidine and active pocket of nsp5 (red) and nsp16 (blue); (iii) Number of hydrogen bond interactions of tomatidine with nsp5 (red) and nsp16 (blue). **D** Docked conformations of tomatidine with PEDV 3CLpro or inactive 3CLpro. The compounds and proteins are represented as sticks and cartoons, respectively. The compounds are colored green. The proteins are colored according to their secondary structures (helix = blue, sheet = purple, loop = pink). The active sites of enzyme pockets are shown as a mesh. **E** The binding energy of the tomatidine–protein complex, calculated using Autodock, is listed.

Several replicative enzymes regulate virus replication [22]. Considering that tomatidine significantly inhibited infection at the virus replication stage, we speculated that tomatidine may work directly with important viral replicative enzymes. To ascertain which replicative enzymes were targeted by tomatidine, potential binding sites were analyzed in detail using Autodock to dock tomatidine into the PEDV replicative enzyme structures (Figure 4A). The estimated binding free energies were ranked as shown in Figure 4B, and the top two complexes (binding energy = − 9.14 kJ/mol, − 8.96 kJ/mol) nsp5 and nsp16 were selected for further molecular dynamics (MD) analysis.

MD utilizes Newtonian physics to simulate atomic movements in a solvated system and is an accurate computational method for simulating protein-drug interactions [23]. To judge the findings of our docking model, a 10 ns molecular dynamic simulation was carried out for the assessment of receptor and ligand stability via Root Mean Square Deviation (RMSD), the distance and the number of hydrogen bonds between tomatidine and the active pockets of the proteins. In tomatidine-nsp5 (3CLpro) simulation, the RMSD values of the backbone converged at less than 0.25 nm and remained stable starting at 7 ns. The higher and more volatility RMSD in tomatidine-nsp16 implied structure instability (Figure 4C(i)). Distances between tomatidine and the active pocket of nsp5 are highly conserved and tight during simulation calculations, whereas those between tomatidine and nsp16 fluctuated wildly (Figure 4C(ii)). We measured the number of hydrogen bonds during the MD simulations to better capture the intermolecular polar interactions. No hydrogen bonds were detected between tomatidine and nsp16. However, tomatidine did form hydrogen bonds with the active pocket of 3CLpro, which may contribute to the stability of the tomatidine-3CLpro complex (Figure 4C(iii)). This means that 3CLpro is a more likely target of tomatidine than nsp16. In addition, the low binding energy of tomatidine to inactive 3CL protease in silico strengthens the possibility that 3CLpro is indeed the target for tomatidine (Figure 4D and E).

### Binding of tomatidine with 3CLpro detected by fluorescence quenching assay and ITC

**Figure 5:**
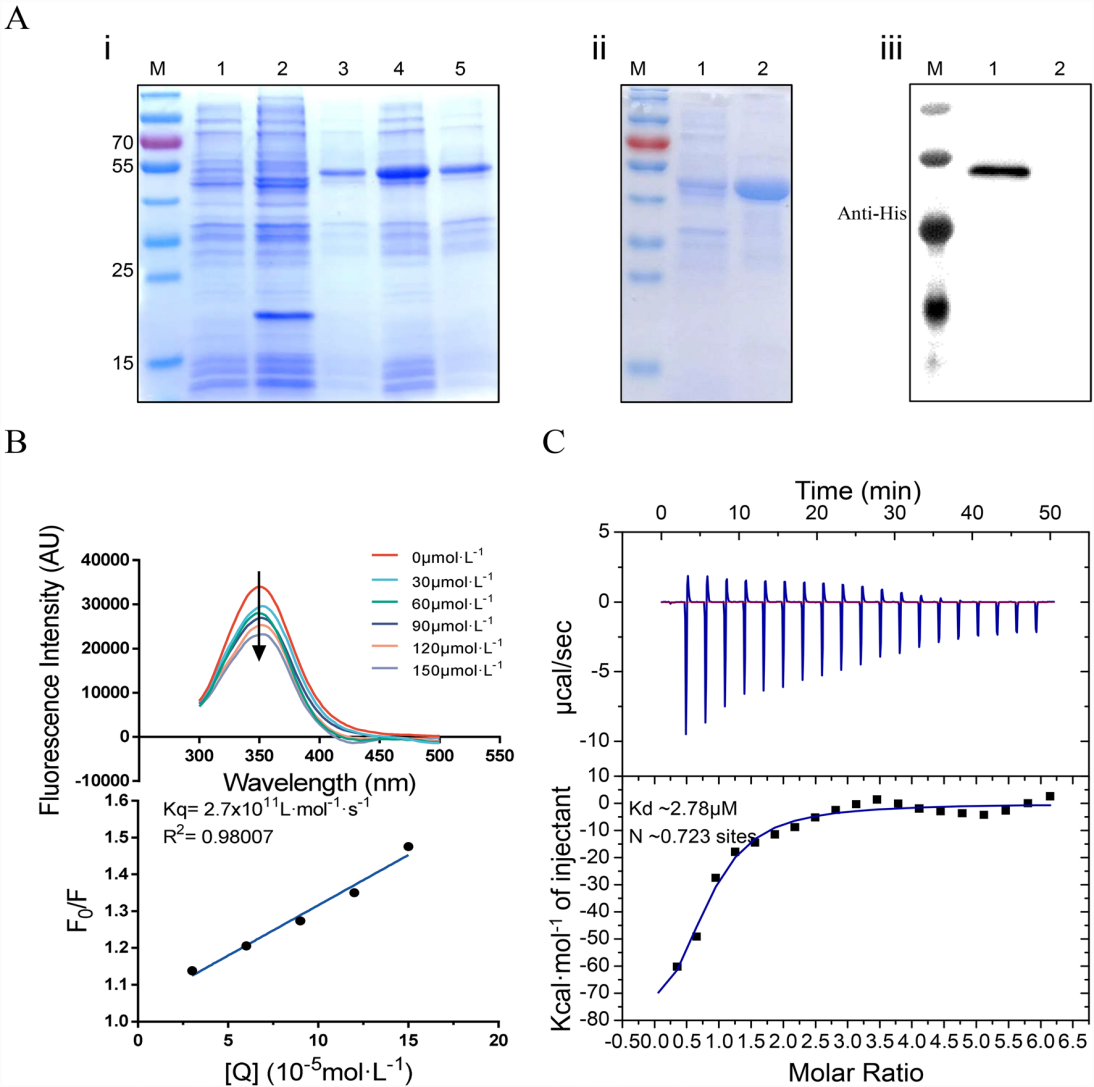
**Binding of tomatidine with 3CLpro detected by fluorescence quenching assay and ITC. A** Expression and purification of recombinant 3CLpro. (i) BL-21 cells transformed with recombinant plasmids pET-32a-3CLpro were subjected to SDS-PAGE. Lane 1, BL21-pET-32a-3CLpro without IPTG induction; Lane 2, BL21-pET-32a with IPTG induction; Lane 3, BL21-pET-32a-3CLpro with IPTG induction; Lane 4, BL21-pET-32a-3CLpro with IPTG induction supernatant after ultrasonication; Lane 5, BL21-pET-32a-3CLpro with IPTG induction precipitation after ultrasonication. (ii) Recombination protein supernatant before (Lane 1) and after (Lane 2) purifications were subjected to SDS-PAGE. (iii) Purified protein was analyzed by Western blot. **B** Upper: Fluorescence emission spectra of different concentrations of tomatidine in the 3CLpro solutions at 50 mg/L; Lower: Stern–Volmer plot describes the 3CLpro quenching caused by association with quencher. **C** Representative thermodynamic profiles of tomatidine binding with the 3CLpro in solution, results of ITC measurements.

To verify the binding of 3CLpro and tomatidine, PEDV 3CLpro fused with His tag was expressed in soluble form using an *E. coli* expression system (Figure 5A(i)). After Ni-Sepharose purification, a single clear target band indicated that the 3CLpro obtained could be used for further experiments (Figure 5A(ii), (iii)). The purified recombinant 3CLpro was then used for a fluorescence quenching assay, ITC and FRET.

A fluorescence quenching assay was carried out to determine the interaction between 3CLpro and tomatidine. The results showed that the fluorescence emission intensity of 3CLpro decreased distinctly in a dose-dependent manner with increasing concentration of tomatidine (Figure 5B upper). The Stern–Volmer plot is shown in Figure 5B lower; the Kq value was calculated to be 2.7 × 10^11^ L·mol^−1^ s^−1^. The maximum scatter collision quenching constant of the various quenchers is 2 × 10^10^ L·mol^−1^ s^−1^. This indicates that the quenching mechanism was static and the interaction between 3CLpro and tomatidine was strong.

The binding affinities of tomatidine with PEDV 3CLpro were measured by ITC, which is widely used to determine thermodynamic parameters of protein–ligand interactions such as the dissociation constant (Kd) and binding stoichiometry (N). The results showed that tomatidine directly interacted with PEDV 3CLpro with a Kd of 2.78 μM and N of 0.723 sites. This result further supports the premise that PEDV 3CLpro is the target protein of tomatidine.

These results allowed us to speculate that tomatidine may block the activity of the 3CLpro, thereby inhibiting PEDV replication.

### Tomatidine directly inhibits the activity of PEDV 3CL protease

**Figure 6:**
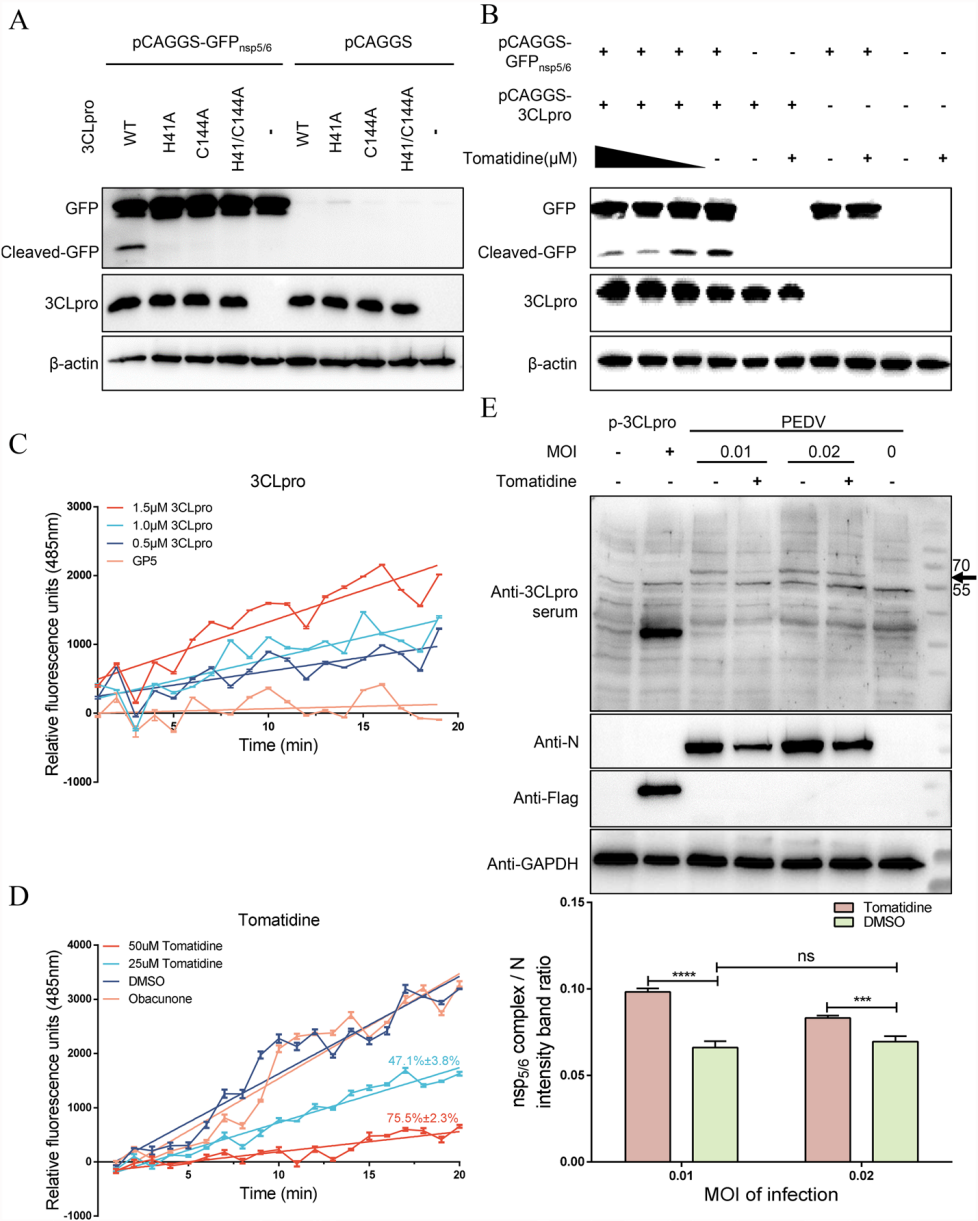
**Tomatidine directly inhibited the activity of PEDV 3CL protease. A** Vero cells cultured in 24-well plates were co-transfected with 250 ng of GFP_nsp5/6_ encoding plasmid, 250 ng of 3CLpro or inactive mutants expressing plasmid or empty vector. At 24 hpi, the cells were harvested and the cleaved fragment of GFP_nsp5/6_ was visualized using western blot. **B** Vero cells cultured in 24-well plates were co-transfected with 250 ng of GFP_nsp5/6_ encoding plasmid, 250 ng of 3CLpro expressing plasmid or empty vector. At 12 hpi, the culture supernatants were replaced with fresh DMEM containing the indicated concentrations (10, 20, and 30 μM) of tomatidine or DMSO. At 24 hpi, the cells were harvested and the cleaved fragment of GFP_nsp5/6_ was visualized using western blot. **C** PEDV 3CLpro was used at final concentrations of 0.5, 1, and 1.5 μM; and 10 μM (3CLpro substrate) was added to the protein in a black 96-well plate. PRRSV GP5 protein was used as a negative control. The mixtures were then further incubated at 37 °C for 20 min, and fluorescence was monitored at 340 nm excitation and 485 nm emissions every minute. The RFU were calculated by subtracting the mock from the fluorescence readings to eliminate the effect of background signals. **D** Tomatidine (25, 50 μM) or obacunone (50 μM, negative control) or DMSO was pre-incubated with 1 μM protease for 20 min at 37 °C, and 10 μM (3CLpro substrate) was added to the mixture in a black 96-well plate. The mixtures were then further incubated at 37 °C for 20 min, and fluorescence intensity was monitored at 340 nm excitation and 485 nm emissions every minute. RFU were calculated as above. **E** Vero cells were inoculated with PEDV (0.01 and 0.02 MOI) and 6 μM tomatidine. Cells were transfected with 250 ng pCAGGS-3CLpro as 3CLpro positive control. After incubation for 16 h, the cellular proteins were collected and the virus polyprotein was detected with western blot using anti-3CLpro mouse antibody, as previously described (20). Meanwhile, PEDV N-protein, GAPDH, and 3CLpro-Flag were detected with western blot using molecular antibodies against PEDV N-protein, GAPDH, and Flag. The ratio of nsp5-6 complex/N protein levels was depicted by integrated density analysis. The arrow indicates the location of nsp5-6 complex. All results are mean ± SD from three independent experiments performed in triplicate.

To explore the impact of tomatidine on 3CLpro activity, we constructed plasmids expressing nsp5 (3CLpro), a series of inactive 3CLpro mutants (H41A, C144A, H41/C144A), and GFP_nsp5/6_ containing the nsp5/nsp6 junction (YGVNLQ^SG) of PEDV. After the Vero cells were co-transfected with these plasmids, western blot assay showed that cleaved fragments of GFP_nsp5/6_ were detected in the presence of 3CLpro, but not in the presence of 3CLpro mutants H41A, C144A, and H41/C144A, indicating that PEDV 3CLpro was active (Figure 6A). When Vero cells were treated with increasing concentrations of tomatidine, the cleaved fragments clearly decreased in a dose-dependent manner (Figure 6B), indicating that tomatidine inhibited the activity of PEDV 3CL protease.

We also used FRET to confirm the effect of tomatidine on 3CLpro activity. The dose-dependent increase of fluorescence intensity shows that the 3CLpro obtained possessed catalytic activity (Figure 6C). Moreover, compared with obacunone, tomatidine significantly inhibited the activity of PEDV 3CLpro (Figure 6D). The percentage of inhibition of 50 μM and 25 μM tomatidine was 75.5% ± 2.3% and 47.1% ± 3.8% respectively, at 20 min incubation. Each reaction was performed in triplicate and the results are expressed as mean ± standard deviation (SD).

In order to explore the inhibition of viral 3CLpro activity of tomatidine in PEDV-infected cells, anti-3CLpro serum antibody was prepared by vaccinating mice with the purified recombinant 3CLpro (Figure 6E, lines 1–2). Vero cells were inoculated with PEDV (0.01 and 0.02 MOI) and 6 μM tomatidine. Western blot showed that the partial virus polyprotein (nsp5-6 complex, rather than 3CLpro) was detected with the anti-3CLpro serum antibody at 16 hpi, as previously described [24]. The ratios of nsp5-6 complex/N in PEDV not treated with tomatidine were similar to each other between the two doses of 0.01 and 0.02 MOI. But the ratios of nsp5-6 complex/N in PEDV treated with tomatidine were significantly higher than those treated with DMSO (Figure 6E, line 3–7), indicating that tomatidine may block the 3CL protease activity in a natural situation.

### Tomatidine has antiviral activity against other swine disease viruses

**Figure 7:**
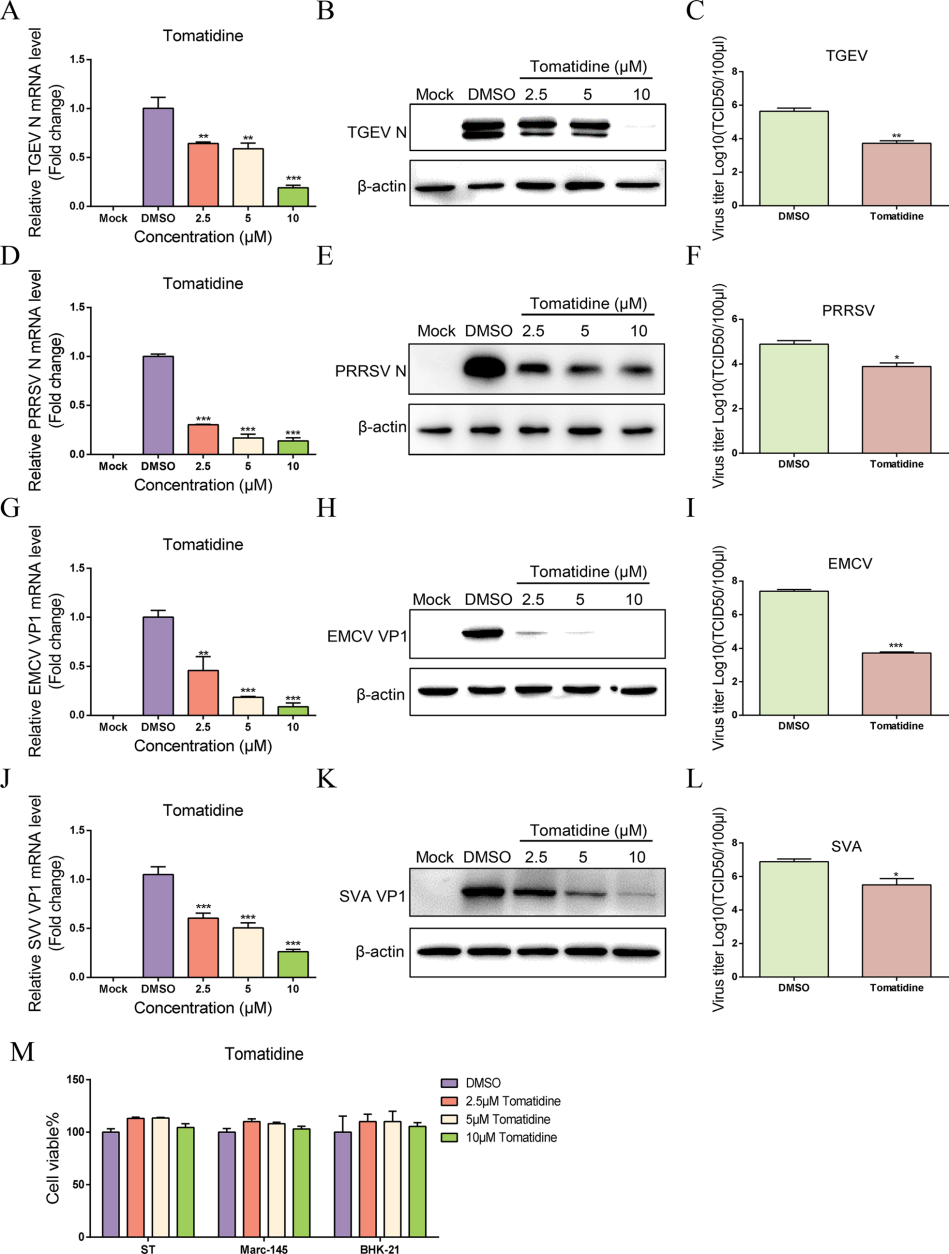
**Tomatidine shows broad-spectrum antiviral activity against other swine disease viruses.** TCID_50_, western blot, and qPCR were used to examine the inhibition activity of tomatidine against other swine disease viruses. Three designated concentrations of compounds were added to the culture medium (final concentrations were 2.5, 5, 10 μM, with no-observable cytotoxicity). DMSO was used as the negative control. TGEV (0.01 MOI), PRRSV (**E**), and EMCV (**H**)/SVA (**K**) were then used to infect ST, Marc-145, and BHK-21 cells, respectively, and samples were harvested at 24 h, 48 h, and 18 h. The TCID_50_ of TGEV (**A**), PRRSV (**D**), EMCV (**G**), and SVA (**J**) treated with 10 μM tomatidine or DMSO were calculated using the Reed-Muench method. The N-protein level of TGEV (**B**), PRRSV (**E**), EMCV (**H**), and SVA (**K**) were determined by western blot. Relative TGEV N (**C**), PRRSV N (**F**), EMCV VP1 (**I**), and SVA VP1 (**L**) mRNA levels, was determined by qRT-PCR, and expressed relative to that in DMSO-treated cells. **M** Viability of ST, Marc-145, and BHK-21 cells pretreated with the indicated concentrations of tomatidine and incubated for 24 h, 48 h, and 18 h, respectively in medium containing tomatidine. The results are from one of three independent experiments. The internal loading control was β-actin. Error bars represent the SD. The asterisks in the figures indicate significant differences (*P < 0.05; **P < 0.01; ***P < 0.001; ns = not significant).

Tomatidine strongly inhibits virus particle production of dengue virus and Chikungunya virus [25, 26]. To investigate whether tomatidine has an antiviral effect against other swine disease viruses, we selected several other viruses including a coronavirus, TGEV, [27], an arterivirus, PRRSV [28], and picornaviruses EMCV and SVA [20, 29, 30]. ST cells were treated with 2.5, 5, and 10 μM tomatidine for 1 h and then infected with TGEV for 24 h. The results indicated that TGEV proliferation was significantly suppressed by tomatidine and almost completely cut off at the concentration of 10 μM (Figure 7A–C). Marc-145 cells were treated with the indicated concentrations of tomatidine for 1 h and infected with PRRSV for 48 h. These results indicated that tomatidine exhibits antiviral activity against PRRSV (Figure 7D–F). BHK-21 cells were treated with the indicated concentrations of tomatidine for 1 h and then infected with EMCV or SVA for 18 h. Western blot, qPCR, and TCID_50_ analysis revealed substantial inhibition activity of tomatidine against EMCV and SVA (Figures 7G–I, and J–L). None of the various cells treated with the indicated concentrations of tomatidine showed any cytotoxicity (Figure 7M).

## Discussion

Outbreaks of porcine epidemic diarrhea cause significant lethality rates in neonatal piglets, creating a heavy economic burden in the global swine industry. Unfortunately, available commercial vaccines fail to protect against the high virulence of PEDV variants [31, 32]. Because there is no antiviral drug available to treat this disease, we need to develop new therapeutic methods to prevent and control PEDV. Natural compounds and compositions have been a rich source of drugs against many viral infections. For example, Griffithsin, a high-mannose-specific lectin, has been shown to reduce PEDV infection in Vero cells by preventing viral attachment and disrupting cell-to-cell transmission [14]. Prenylated phenolic compounds from the leaves of *Sabia limoniacea* exhibit promising antiviral activities against PEDV replication [16]. But the mechanisms involved in these studies are not well understood. In this study, tomatidine, screened from a library of 911 natural products, was shown to inhibit PEDV proliferation in vitro. Tomatidine displayed remarkable inhibition of PEDV replication by targeting 3CL protease, which we investigated using molecular docking and MD analysis, fluorescence spectroscopy, ITC, 3CL protease activity, and FRET assays.

The PEDV life cycle is composed of four stages: attachment, entry, replication, and release [33]. Our results indicate that tomatidine significantly inhibits viral infection at the replication stage. Several non-structure proteins are key to viral replication, hence we speculated that tomatidine, which is shown to inhibit viral replication, may target a non-structural protein. Making use of molecular docking and molecular dynamics, we speculated that tomatidine may inhibit PEDV proliferation by directly targeting 3CL protease. Fluorescence quenching is an important technique for measuring binding affinity between ligands and proteins. ITC is a technique used in a wide variety of quantitative studies of biomolecular interactions. ITC works by directly measuring the heat that is either released or absorbed during a biomolecular binding event. In our study, 3CLpro-tomatidine binding was indicated by fluorescence quenching and ITC. In order to confirm the effect of tomatidine on 3CLpro activity, we constructed a plasmid expressing GFP_nsp5/6_ protein containing the nsp5/nsp6 junction (YGVNLQ^SG) of PEDV. Our results show that GFP_nsp5/6_ was cleaved into two fragments by 3CLpro, indicating that PEDV 3CLpro was active. Tomatidine inhibited the activity of PEDV 3CL protease in Vero cells in a dose-dependent manner. The inhibition ratio of tomatidine was also shown in a FRET assay. The 485 nm fluorescence intensity remained stable until the labeled substrate was fractured by the protease, which indicates that the purified protein possesses 3CL protease activity. Compared with the control drug obacunone, tomatidine significantly reduced the fluorescence intensity. The ratio of nsp5-6 complex/N in virus treated with tomatidine was significantly higher than in the DMSO treated virus, indicating that tomatidine blocked the 3CL protease activity in a natural situation. However, anti-3CLpro serum raised in mice detected nsp5-6 complex rather than 3CLpro (nsp5) only. This phenomenon underlines the possibility that there is a quantitative difference between nsp5-6 complex and nsp5 of products in PEDV-infected cells at 16 hpi.

The 3C-like protease, which is responsible for processing polyproteins of nidoviruses and picornaviruses, is an attractive target for drug therapy. It has been reported that a series of compounds such as flavonoids, chalcones, and benzothiazolium display significant antivirus activity by inhibiting 3CLpro activity. We found that tomatidine strongly inhibits PEDV replication by targeting 3CL protease. Tomatidine has many cell biological properties. It enhances expression of nuclear factor erythroid 2-related factor 2 (Nrf2) [34], which when knocked out benefits viral propagation [19]. 5′-AMP-activated protein kinase catalytic subunit alpha-2 (AMPK) promotes innate immunity and antiviral defense through modulation of STING signaling [35]. Tomatidine also stimulated AMPK phosphorylation by activating the CaMKKβ pathway [36]. Tomatidine inhibits dengue virus particle production partly by inhibiting cyclic AMP-dependent transcription factor ATF-4 (ATF4) [26]. In this study, we noted that the IC_50_ (50% inhibitory concentration) of tomatidine against PEDV in cells was low micromolar (3.4 μM); the CC_50_ (50% cytotoxic concentration) was about 45.68 μM; while the inhibition of PEDV 3CLpro by tomatidine in the FRET assay at 50 μM was only 75%. In addition, the binding energy of tomatidine with the viral nsp16 was close to that of the 3CLpro. These results suggest that there may be other antiviral mechanisms involved. 3CLpro of PEDV, as well as of other coronaviruses (Porcine deltacoronavirus), picornaviruses (Hepatitis A virus, foot and mouth disease virus), and arterivirus (PRRSV), has been shown to inhibit production of INF-β, hijacking the host’s innate antiviral immune response [37–40]. This effect has been attributed to the direct cleavage of NF-kappa-B essential modulator (NEMO) (an important substrate in the RLR cascade) [41, 42]. By blocking 3CLpro, tomatidine may also prevent 3CLpro cleaving NEMO, thereby enhancing the innate antiviral immunity, which could in turn help to inhibit virus replication.

TGEV, PRRSV, EMCV, and SVA contain 3C or 3CL protease. Our results showed that tomatidine effectively inhibits TGEV, PRRSV, EMCV, and SVA replication in vitro. But the antiviral activity of tomatidine against picornaviridae looks more significant than TGEV and PRRSV. It may be related to the different characterization of the virus replication. The molecular docking analysis demonstrated that the binding energy between the 3C or 3CL protease of the viruses with tomatidine were weak and no hydrogen bonds were detected, which being different from that of PEDV 3CLpro (Additional file 1). It indicates that the antiviral activity of tomatidine is likely to rely on other pathways rather than only targeting 3C proteases, which is worthy being further explored.

Tomatidine has been proved to be safe within a certain dosage in mice. Unfortunately, as a dietary supplement approved by the FDA, tomatidine lacks detailed safety and pharmacokinetics evaluation in pigs. The safety, pharmacokinetics, and efficacy of tomatidine for prevention, treatment, and control of PEDV and perhaps other porcine nidoviruses need to be further evaluated in pigs. It would be interesting to test whether its 3CLpro blocking activity also holds for nidoviruses infecting humans.

We conclude that tomatidine effectively inhibits PEDV proliferation via inhibition of 3CLpro activity. In addition, tomatidine displays antiviral activity against TGEV, PRRSV, EMCV, and SVA. These findings offer novel and promising therapeutic possibilities for fighting infections caused by these viruses.

## Supplementary information

**Additional file 1. The binding energy of tomatidine to 3CL or 3C protease of TGEV, PRRSV, EMCV, and SVA in silico.** **A** Docked conformations of tomatidine with 3CL or 3C protease of TGEV, PRRSV, EMCV, and SVA in silico. The compounds and proteins are represented as sticks and cartoons, respectively. The compounds are colored green. The proteins are colored according to their secondary structures (helix = blue, sheet = purple, loop = pink). The active sites of enzyme pockets are shown as a mesh. **B** The binding energy of the tomatidine–protein complex, calculated using Autodock, is listed.

## Data Availability

Not applicable.
