# Supplementary figures and images for: Tomatidine inhibits porcine epidemic diarrhea virus replication by targeting 3CL protease
Source: Vet Res. 2020 Nov 11;51:136. doi: 10.1186/s13567-020-00865-y (PMC7656508; doi:10.1186/s13567-020-00865-y)

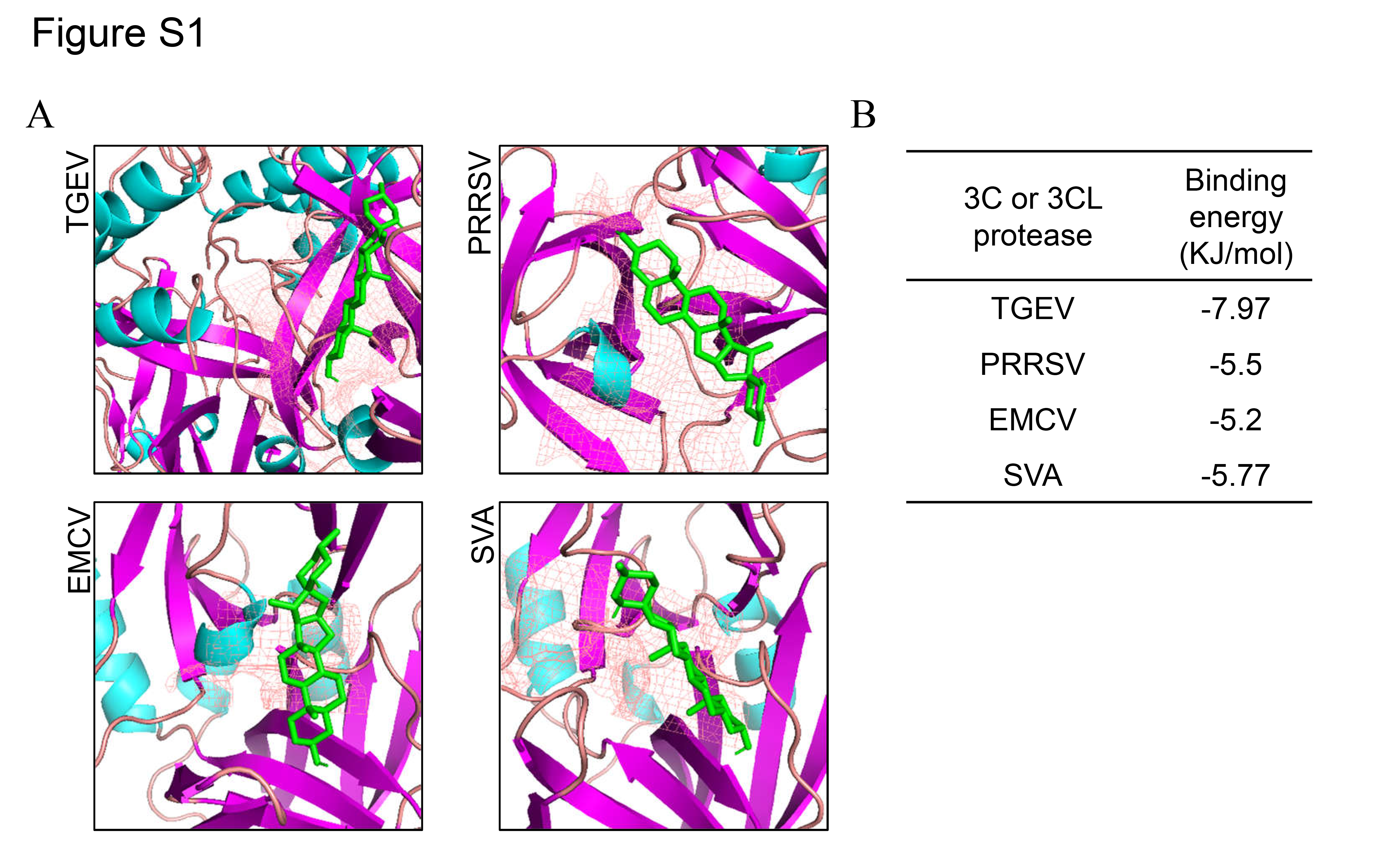

Supplement: Supplementary file 1 — Additional file 1. The binding energy of tomatidine to 3CL or 3C protease of TGEV, PRRSV, EMCV, and SVA in silico. A Docked conformations of tomatidine with 3CL or 3C protease of TGEV, PRRSV, EMCV, and SVA in silico. The compounds and proteins are represented as sticks and cartoons, respectively. The compounds are colored green. The proteins are colored according to their secondary structures (helix = blue, sheet = purple, loop = pink). The active sites of enzyme pockets are shown as a mesh. B The binding energy of the tomatidine–protein complex, calculated using Autodock, is listed. [file 13567_2020_865_MOESM1_ESM.tif]
